# Repetitive Elements in *Mycoplasma hyopneumoniae* Transcriptional Regulation

**DOI:** 10.1371/journal.pone.0168626

**Published:** 2016-12-22

**Authors:** Amanda Malvessi Cattani, Franciele Maboni Siqueira, Rafael Lucas Muniz Guedes, Irene Silveira Schrank

**Affiliations:** 1 Centro de Biotecnologia, Programa de Pós-Graduação em Biologia Celular e Molecular, Universidade Federal do Rio Grande do Sul (UFRGS), Porto Alegre, Rio Grande do Sul, Brazil; 2 Laboratório de Bioinformática, Laboratório Nacional de Computação Científica (LNCC), Petrópolis, Rio de Janeiro, Brazil; 3 Centro de Biotecnologia, Departamento de Biologia Molecular e Biotecnologia, Instituto de Biociências, Universidade Federal do Rio Grande do Sul (UFRGS), Porto Alegre, Rio Grande do Sul, Brazil; Miami University, UNITED STATES

## Abstract

Transcriptional regulation, a multiple-step process, is still poorly understood in the important pig pathogen *Mycoplasma hyopneumoniae*. Basic motifs like promoters and terminators have already been described, but no other cis-regulatory elements have been found. DNA repeat sequences have been shown to be an interesting potential source of cis-regulatory elements. In this work, a genome-wide search for tandem and palindromic repetitive elements was performed in the intergenic regions of all coding sequences from *M*. *hyopneumoniae* strain 7448. Computational analysis demonstrated the presence of 144 tandem repeats and 1,171 palindromic elements. The DNA repeat sequences were distributed within the 5’ upstream regions of 86% of transcriptional units of *M*. *hyopneumoniae* strain 7448. Comparative analysis between distinct repetitive sequences found in related mycoplasma genomes demonstrated different percentages of conservation among pathogenic and nonpathogenic strains. qPCR assays revealed differential expression among genes showing variable numbers of repetitive elements. In addition, repeats found in 206 genes already described to be differentially regulated under different culture conditions of *M*. *hyopneumoniae* strain 232 showed almost 80% conservation in relation to *M*. *hyopneumoniae* strain 7448 repeats. Altogether, these findings suggest a potential regulatory role of tandem and palindromic DNA repeats in the *M*. *hyopneumoniae* transcriptional profile.

## Introduction

*Mycoplasma hyopneumoniae* is a diminutive bacterium, characterized by a small genome (0.92 Mb) with a low GC content [1]. It is commonly associated with mycoplasmal pneumonia in pigs [2] and infected animals are affected by a sporadic, dry and non-productive cough, retarded growth rate and inefficient utilization of feed [3]. Until now, the genomes of six strains of *M*. *hyopneumoniae* have been sequenced [1, 4–7] and the availability of their sequences has enabled whole comparative genome analyses [8]. Transcription, a multi-step mechanism, is finely regulated in all forms of life and is still poorly understood in *M*. *hyopneumoniae*. The occurrence of transcription units [4, 9, 10], promoters [11, 12] and terminators [13] has already been described in this species, but the existence of other regulatory sequences remains to be elucidated.

DNA repeats, widespread and characterized in eukaryote genomes, can also play an important role in prokaryote genomic regulation [14]. It is usually hypothesized that repeats arise by successive duplications and several causal mechanisms, like homologous recombination, slipped-strand mispairing of DNA polymerase or by events of genomic transposition [15]. Bacterial repeats are commonly classified as low-complexity repeats and longer repeats. Low-complexity repeats can be composed of simple oligonucleotides (typically ranging from mononucleotide to pentanucleotide in size) repeated several times in a head-to-tail configuration (tandem), while longer repeats may include complex and spaced repeats, such as palindromes, transposable elements, inverted sequences, minisatellites and large tandem repeats spread throughout the genome [16].

DNA repeats can be found within coding-sequences (CDS), in intergenic regions, or in transposable elements, reflecting both regulatory and structural requirements for the bacterial chromosome [17, 18]. Phase variation, an important mechanism of evolution and adaptation of prokaryotes, is capable of switches in an ON/OFF way the expression of important genes. The presence of repetitive elements, especially simple sequence repeats (SSR) is the major source of variability in these cases. Mutations in SSRs within the coding region can be related to the shifting of the reading frame of proteins. Mutations in noncoding or promoter-located SSRs generate more subtle variations in protein expression levels due to alterations in the relative spacing and positional orientation of promoter elements [18, 19]. The presence of repetitive elements within intergenic regions has already been related to pathogenicity through phase variation in *Neisseria* species, *Haemophilus parainfluenzae*, *Moraxella catarrhalis* [20] and *Mycoplasma hyorhinis* [18]. Many other phenotypes associated with repetitive genotypes were reviewed by Belkum et al. [14] involving microbial evolution, pathogenesis and molecular epidemiology. In *Mycoplasma* spp., the presence of some repetitive elements has been described in *Mycoplasma genitalium* [21–24], *Mycoplasma gallisepticum* [23, 25, 26], *Mycoplasma bovis* [27], *M*. *hyorhinis* [28], *Mycoplasma fermentans* [29], *Mycoplasma synoviae* [23, 30], *Mycoplasma pneumoniae* [23, 24, 31], *Mycoplasma hominis* [32], *Mycoplasma penetrans* [23], *Mycoplasma pulmonis* [23, 24, 33], *Mycoplasma mobile* [23] and *Mycoplasma mycoides* [23, 34] genomes. In *M*. *hyopneumoniae* the presence of SSR elements [23] and some specific palindromic repeats [35] have already been reported, however a genome-wide approach correlating DNA repeats with their possible genomic regulatory consequences is not yet available. Therefore, in this work, an *in silico* prediction associated with experimental validation was performed, aiming to verify and associate the presence of tandem and palindromic repeats as transcriptional regulatory sites.

## Materials and Methods

### DNA repeats *in silico* analysis

Based on the most common repeats already described in prokaryote genomes, especially in *Mycoplasma* spp. [36], two types of DNA repeats were selected to be investigated in this work (tandem and palindromic elements). Tandem sequences were divided into the following: i) Simple Sequence Repeats (SSR); ii) Simple Sequence Repeats of Mononucleotides (SSRM) and iii) Direct Repeats (DR). Palindromic elements were searched using two approaches: allowing gaps (PALG) or not allowing gaps (PAL). In-house PERL scripts were used to extract intergenic regions up to 500 bp upstream from the start codon of all *M*. *hyopneumoniae* strain 7448 (NSDC AE017244.1) CDSs which were used as input for the software’s prediction. The annotation of the genome was manually cured, with confident translational start codons. For each type of repeat sequence, two independent software packages were used, as detailed below.

In the present work, SSR is defined as a sequence containing 2 to 10 repeated nucleotides as described by Huang et al. [36]. The algorithms used to search for SSR were SSRLocator [37] and Simple Sequence Repeat Identification Tool (SSRIT) [38].

SSRM is defined as a mononucleotide repeated at least 8 times in a head-to-tail orientation. The software packages used to predict SSRM were Tandem Repeat Finder (TRF) [39] and SSRIT. Only perfect repeats of SSR and SSRM were analysed, once mismatches were not allowed in this approach.

DR is defined as an element with 11 to 50 nucleotides repeated at least twice in a tandem way. TRF and ETANDEM from The European Molecular Biology Open Software Suite (EMBOSS) [40] were used to predict this type of repeat. In this case, output data from TRF and ETANDEM were filtered to include only DR repeats that had identity of ≥95% within the repeated copies.

PALG is an inverted repeat of 9 to 20 nucleotides containing additional gaps ranging from 5 to 15 bases. Palindrome from EMBOSS and Palindrome Search [41] were the algorithms used in this approach.

PAL is an inverted repeat of 9 to 15 bases with no gaps. The same software was used to predict PAL and PALG, and only elements that had the maximum of 2 mismatches between the inverted repeat were considered.

Just PALG and PAL elements that were located up to 300 bp distant from the ATG were analyzed, the remaining data was excluded. After the individual search of each element, the results were grouped and all overlaps were eliminated according to the following hierarchy: PAL, SSRM, DR, SSR and PALG.

All repetitive elements found in noncoding regions of *M*. *hyopneumoniae* strain 7448 were classified based on the downstream CDS, ATG distance, association with promoter sequences and transcription unit distribution.

**Fig 1:**
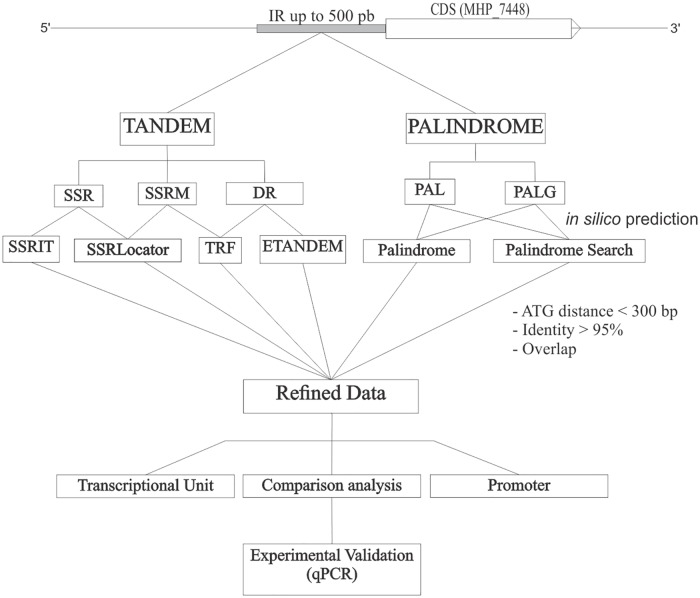
Pipeline of repeat search strategy. Up to 500 bp of 5’ intergenic regions (IR) of all *M*. *hyopneumoniae* strain 7448 (MHP_7448) CDSs were used for *in silico* prediction of tandem and palindromic DNA repeats.

A search for common motifs among repeat classes was performed through the web server MEME SUITE [42], using default parameters. A complete workflow of the search strategy was represented in Fig 1.

### Comparative analysis

**Fig 2:**
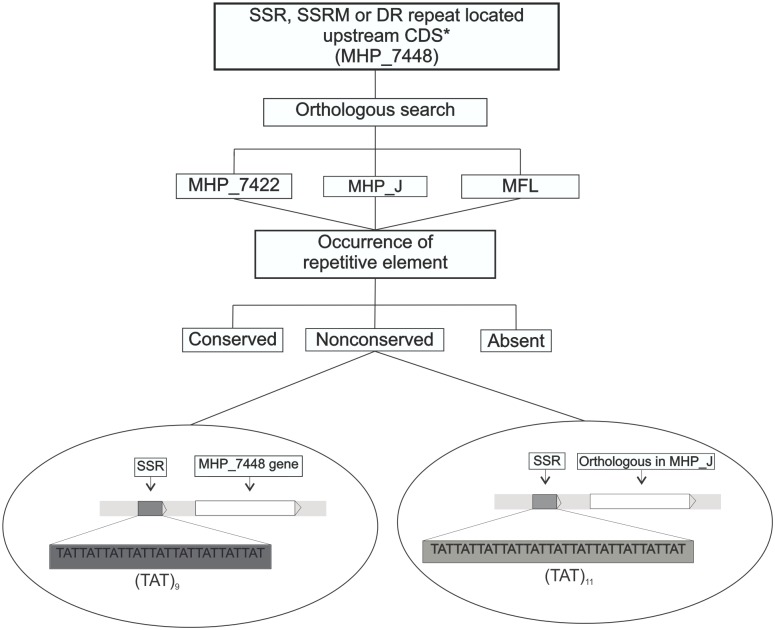
Pipeline of tandem repeat comparison analysis among mycoplasma genomes. An example of nonconserved SSR between *M*. *hyopneumoniae* strain 7448 (MHP_7448), and *M*. *hyopneumoniae* strain J (MHP_J) can be observed above. A (TAT)_9_ repeat found in the MHP_7448 gene intergenic region was classified as nonconserved, as a (TAT)_11_ repeat was localized in the respective orthologous intergenic region in MHP_J. Abbreviations: *M*. *hyopneumoniae* strain 7422 (MHP_7422); *M*. *flocculare* (MFL). *First CDS of the transcription unit.

To validate the relevance of repetitive elements found, comparison analysis was performed using three different approaches. Initially, all tandem repeats (SSR, SSRM and DR) found upstream of the first gene of *M*. *hyopneumoniae* strain 7448 transcription units [9, 10] were compared against three other mycoplasma genomes: *M*. *hyopneumoniae* strain 7422 –pathogenic; *M*. *hyopneumoniae* strain J–nonpathogenic; and *Mycoplasma flocculare* ATCC 27716 –phylogenetically related to *M*. *hyopneumoniae* and nonpathogenic. Therefore, a BLAST [43] search was performed with the first CDS of each transcription unit against the genomes of the three organisms mentioned above. The corresponding tandem motif was manually localized in the 5´upstream region of each orthologous gene. One of three classifications could be assigned to each repeat: conserved (C)–the repetitive element was exactly the same in sequence and number; nonconserved (NC)–the copy number of the tandem element was different; and absent (A)–the repetitive element was not found in the orthologous gene. A schematic pipeline of the tandem comparison can be seen in Fig 2.

**Fig 3:**
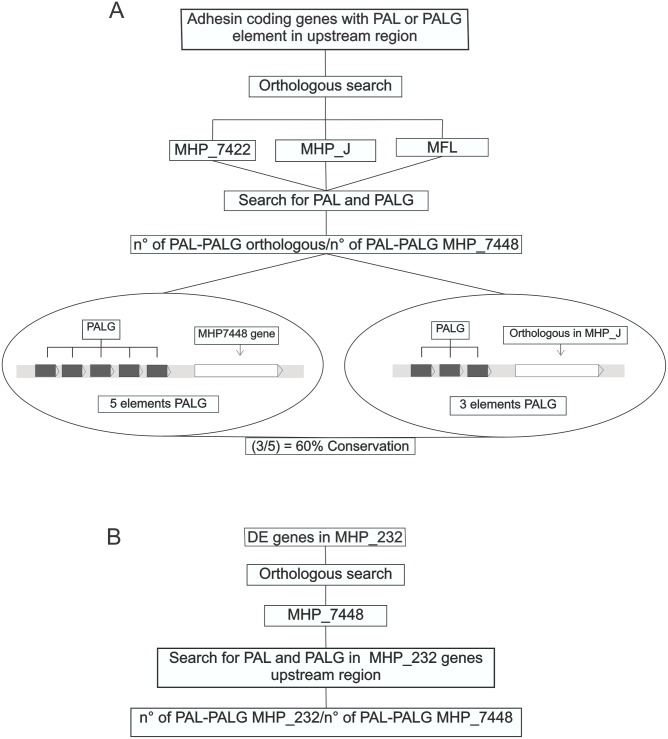
Pipeline of repeat conservation in putative adhesin-coding genes and differentially expressed genes from strains of *M*. *hyopneumoniae*. (A) Comparison analysis of adhesins, with an example of relative conservation between MHP_7448 genes that have 5 PALG repeats and the respective orthologues in MHP_J that have only 3. The percentage (%) conservation between MHP_7448 and MHP_J repeats was obtained by dividing the number of repeat elements. (B) Repeats present in differentially expressed (DE) genes found in *M*. *hyopneumoniae* strain 232 (MHP_232) were compared with those found in MHP_7448 in the same way as in (A). Abbreviations as in Fig 2.

The second comparison analysis was performed in genes coding for putative adhesins [44]. Each palindromic repeat (PAL or PALG) already found upstream of the start codon of adhesin-coding genes in *M*. *hyopneumoniae* strain 7448 were compared among the same mycoplasma genomes previously described. The repeat search was conducted directly upstream of the region of the orthologous gene or in the upstream region of the corresponding transcription unit. The comparison of PAL and PALG among mycoplasmas genomes were performed by Pattern Locator software [45]. Differently from the tandem repeat strategy, just two classifications could be assigned to the PALG or PAL elements: 1) conserved–the inverted repeat that composed the palindromic element is similar (maximum of 3 mismatches) to that found in *M*. *hyopneumoniae* strain 7448 or 2) absent–the repetitive element PALG or PAL was not identified. The level of conservation (%) of palindromic repeats between mycoplasma genomes was calculated by dividing the number of conserved repeats found in the noncoding region of orthologue gene by the total number of repeats found in the respective *M*. *hyopneumoniae* strain 7448 gene (Fig 3A).

The third approach was based on previous studies of *M*. *hyopneumoniae* strain 232, in which microarray assays have identified genes that are differentially expressed in some growing adverse situations, such as exposure to norepinephrine [46] or hydrogen peroxide [47], *in vitro* infection [48], iron depletion [49] and heat shock [50]. Using BLAST search [43] orthologous of the differentially expressed genes from *M*. *hyopneumoniae* strain 232 were found in *M*. *hyopneumoniae* strain 7448 genome. A comparison analysis of palindromic repeats that were predicted in *M*. *hyopneumoniae* strain 7448 against *M*. *hyopneumoniae* strain 232 was performed as described for the adhesin genes search strategy (Fig 3B). As palindromic elements can form secondary structure, the ΔG was evaluated by QuickFold algorithm, [51] using default parameters.

### qPCR experiments

Culture conditions, RNA isolation and cDNA synthesis by Reverse Transcription (RT) were performed as described in Siqueira et al. [10]. Target and primer descriptions for *M*. *hyopneumoniae* strain 7448, *M*. *hyopneumoniae* strain J and *M*. *flocculare* ATCC 27716 reactions are available in S1 Table. Primers were designed using *Vector NTI Advance 10* (Invitrogen, USA).

Quantitative PCR (qPCR) assay was performed using 1:50 cDNA as template and Platinum SYBR Green qPCR SuperMix-UDG (Invitrogen, USA) on 7500 Real-Time PCR Systems (Applied Biosystems, USA). The qPCR reactions were carried out at 90°C for 2 min and 95°C for 10 min followed by 40 cycles of 95°C for 15 s and 60°C for 1 min. A melting curve analysis for each primer pair was done to verify primer efficiency.

Relative expression of each gene was evaluated in *M*. *hyopneumoniae* strain 7448, *M*. *hyopneumoniae* strain J and *M*. *flocculare* ATCC 27716 RNAs. Relative expression of mRNA was calculated by the 2^-ΔCt^ method [52]. The threshold cycle (CT) values were normalized to the reference gene *lon* (MHP7448_0524). Reference gene was determined in a specific assay where expression profiles of several genes were evaluated in the three mycoplasma RNAs tested (*M*. *hyopneumoniae* strain 7448, *M*. *hyopneumoniae* strain J and *M*. *flocculare* ATCC 27716). The gene that showed no differential expression in all mycoplasma genomes tested was used in relative expression calculations. Three technical and two biological replicates were done for each target evaluated. Statistical analyses were performed using GraphPad Prism 6 software by One-way ANOVA followed by Tukey’s multiple comparison test (P < 0.05).

## Results

### *In silico* prediction

Computational analysis predicted the presence of 340 tandem repeats and 1,879 palindromic elements in the genome of *M*. *hyopneumoniae* strain 7448. Among the tandem repeats, 272 SSRM, 55 SSR and 13 DR repeats were found. Palindromic elements were divided into 689 PAL and 1,190 PALG (Table 1). These results were further analysed and all overlapping sequences were excluded. We could observed that PAL elements, in most of the cases, overlapped partially with PALG (Table 1) and therefore PAL overlapping elements were excluded from further analysis. The decision to exclude was based on the capability of the selected PALG elements to form stronger secondary structure, representing an interesting physical modification in the DNA molecule [53]. As a result, 59% of previously located repeats were maintained in the *M*. *hyopneumoniae* strain 7448 genome, distributed as 144 tandem (111 SSRM, 29 SSR and 4 DR repeats) and 1,171 palindromic repeats (73 PAL and 1,098 PALG) (Table 1; S2 Table). The presence of the tandem repeats and palindromic elements was confirmed by utilization of two independent software packages. The best results were found with the SSR repeats wherein almost 90% of elements found were predicted by both algorithms tested (Table 1).

**Table 1:** Computational analysis of tandem and palindromic repeats in the *M*. *hyopneumoniae* strain 7448 genome.

Repeats	Software	General Data		Refined Data	
		N° repeats	N° CDS*	N° repeats	N° CDS*
**SSRM**	TRF	1	1	0	0
	SSRLocator	229	165	82	68
	TRF x SSRLocator^a^	42	41	29	29
	**Total**	**272**	**192**	**111**	**89**
**SSR**	SSRIT	5	4	2	2
	SSRLocator	3	3	1	1
	SSRIT x SSRLocator^b^	47	44	26	25
	**Total**	**55**	**48**	**29**	**28**
**DR**	TFR	4	4	2	2
	Etandem	8	8	1	1
	TRF x etandem^c^	1	1	1	1
	**Total**	**13**	**13**	**4**	**4**
**PAL**	Palindrome	226	169	26	25
	Palindrome Search	244	174	27	26
	Palindrome x Palindrome Search^d^	219	169	20	19
	**Total**	**689**	**314**	**73**	**62**
**PALG**	Palindrome	843	336	779	321
	Palindrome Search	168	134	149	119
	Palindrome x Palindrome Search^e^	179	138	170	132
	**Total**	**1190**	**393**	**1098**	**373**

General data represent independent software prediction. Refined data show filtered sequences, without overlaps.

*Number of CDS found downstream the predicted repeats.

^a^SSRM repeats that were predicted by the both software (TRF and SSRLocator).

^b^SSR repeats that were predicted by the both software (SSRIT and SSRLocator).

^c^DR repeats that were predicted by the both software (TRF and etandem)

^d^PAL and ^e^PALG predicted by the both software (Palindrome and Palindrome Search).

The distribution of repetitive elements among 5´upstream regions of *M*. *hyopneumoniae* strain 7448 CDSs demonstrated an average of 3 PALG, 1 PAL, 1 SSRM, 1 SSR and 1 DR repeat per 5’ upstream region of a unique CDS (Table 1). Moreover, different combinations of palindrome and tandem repeats could be observed in a single intergenic region (S2 Table). Comparative analysis of nucleotide sequences in each class of repetitive element was unable to determine a common motif for tandem repeat or palindromic sequence, as they were diverse in nucleotide composition and length (S2 Table).

### Repeat Classification

**Fig 4:**
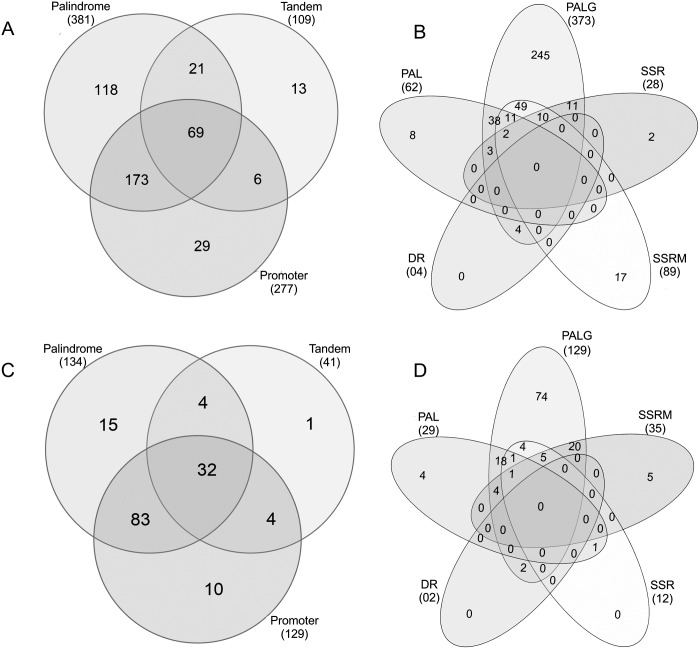
Repeat distribution in the intergenic regions of *M*. *hyopneumoniae* strain 7448. (A) All intergenic regions of the genes were analysed in relation to the presence of tandem repeats, palindromic sequences or putative promoter motifs. (B) Tandem repeats and palindromic sequences were distributed in SSR, SSRM, DR, PAL and PALG throughout genes intergenic regions. The same was done for the 5’ upstream region of the first gene of each transcription unit described in genome (C) and (D).

All 730 genes and 162 transcriptional units (polycistronic and monocistronic) [10] of *M*. *hyopneumoniae* strain 7448 were classified according to the cis-elements found in the upstream region of the respective start codon. Analyses of all genes revealed that 429 of them (59%) had putative promoter sequences (according to Weber et al. [12]) or repetitive element (present analysis) in the 5’ upstream region. Among these 429 genes, the most representative element in the 5’ upstream region were palindromic repeats plus promoter sequences (40%), followed by individual palindromic repeats (28%) and the combination of all putative sequences (tandem, palindromic and promoter) representing 16% (Fig 4A).

The distribution of tandem and palindromic elements showed that individually, PALG were present in the majority of the genes 5’ upstream regions investigated (57%). The combination of PALG plus SSRM repeats was seen in 11% of genes 5’ upstream regions. PALG plus PAL elements were seen in 9% of genes 5’ upstream regions. None of the genes had exclusively DR repeats or a combination of all repeat types in a single 5’ upstream region (Fig 4B).

Considering all 162 transcriptional units of *M*. *hyopneumoniae* strain 7448, 149 (92%) of them contained a putative regulatory element (repeat or promoter) in the respective 5’ upstream region from the start gene of the transcription unit. Among the 149 transcription units, 56% contained palindromic elements associated with putative promoters; 21% showed the presence of the three elements (tandem, palindrome and promoter), and 10% contained only palindromic sequences. The three situations mentioned above represented 87% of all possible cases of elements in the regulatory regions of the transcriptional units (Fig 4C). The classification of palindromic sequences and tandem repeats in the 5’ region of the transcription units resulted in 50% of transcription units with PALG, 13% with PALG plus SSRM and 12% having PAL plus PALG. None of the transcription unit 5’ upstream regions showed the presence of an exclusive DR or SSR element, and finally, none possessed the five elements together (Fig 4D).

### Comparative analysis: conservation of repeat elements in orthologous CDSs

Aiming to understand the prevalence of distribution of the repeat elements identified in *M*. *hyopneumoniae* strain 7448, a comparative analysis was performed with other swine *Mycoplasma* species. In this work, the presence of repeat sequence in the 5’ upstream regions of orthologous genes in *M*. *hyopneumoniae* strain 7422 (pathogenic) and *M*. *hyopneumoniae* strain J (nonpathogenic), and also with the phylogenetically related nonpathogenic mycoplasma species, *M*. *flocculare*, was explored (see Fig 2 for the pipeline analysis).

A total of 45 genes (S3 Table) that presented tandem repeats (SSR, SSRM or DR) in their 5’ upstream regions and were positioned as the first gene of a transcriptional unit were selected for comparison analysis. Concerning the 45 *M*. *hyopneumoniae* strain 7448 genes investigated by the BLAST approach, 97%, 90% and 67% of them displayed orthologous genes in the genome of *M*. *hyopneumoniae* strain 7422, *M*. *hyopneumoniae* strain J and *M*. *flocculare*, respectively. Repeat comparison analysis was performed only among the orthologous genes found in all genomes and localized in the upstream region of the first gene in each transcription unit. Therefore, 4 DR repeats, 13 SSR and 44 SSRM elements were selected for further comparative studies.

**Fig 5:**
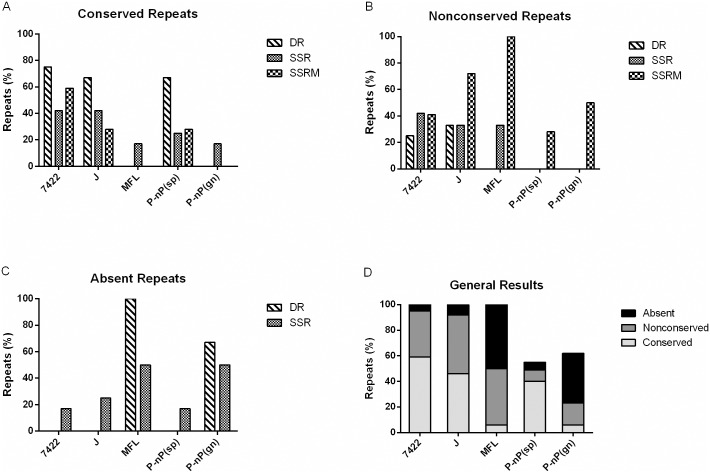
Conservation of repetitive elements in *Mycoplasma* genomes. Graphics represent percent of relative conservation (%) of conserved (A), nonconserved (B) and absent (C) SSR, SSRM, and DR repeats found in *M*. *hyopneumoniae* strain 7448 intergenic regions in relation to strains 7422, J and *M*. *flocculare* (MFL) orthologues. A pathogenic versus nonpathogenic comparison was done and defined as P-nP. Repeats conserved between *M*. *hyopneumoniae* strain 7448 and *M*. *hyopneumoniae* strain 7422 were investigated in relation to MHP_J (P-nP (sp)) and MFL (P-nP(gn)). (D) Graphic represents the average of all tandem repeats found in each situation tested.

As expected, detailed analysis revealed that conservation in repeat sequences among orthologous genes was higher between strains of the same species (*M*. *hyopneumoniae* strain 7422 and *M*. *hyopneumoniae* strain J) than between different species (*M*. *hyopneumoniae* and *M*. *flocculare*) (Fig 5A; S3 Table). However, differences were found between *M*. *hyopneumoniae* strain 7448 versus *M*. *hyopneumoniae* strain 7422 and *M*. *hyopneumoniae* strain 7448 versus *M*. *hyopneumoniae* strain J when each repetitive element was analysed. Greater differences could be seen in SSRM elements, which are 60% conserved in *M*. *hyopneumoniae* strain 7422, 30% in *M*. *hyopneumoniae* strain J and had no conserved elements in *M*. *flocculare*. Interestingly, although the number of repetitive elements classified as “nonconserved” maintained the same pattern previously observed for strains of *M*. *hyopneumoniae*, it increased during analysis between *M*. *hyopneumoniae* strain 7448 and *M*. *flocculare* orthologous genes. DR and SSR showed lower values of divergence in copy number (nonconserved) compared with SSRM (see Fig 5B). Also, the number of orthologues that did not share repetitive elements with *M*. *hyopneumoniae* strain 7448 was higher in the *M*. *flocculare* genome, mainly within the DR repeat class, which was completely absent. Lower absence values were seen in SSR repeats compared with DR repeats and none SSRM were absent in the tested situation (see Fig 5C).

To understand the relation between the presence of repetitive elements and pathogenicity, a further comparative analysis was performed considering the following: repeats must be identical in *M*. *hyopneumoniae* strain 7448 and *M*. *hyopneumoniae* strain 7422 orthologous genes and only those elements that fitted this feature were analysed in relation to *M*. *hyopneumoniae* strain J or *M*. *flocculare* orthologues. These comparisons were named “Pathogenic-non-Pathogenic species” (P-nP(sp)) and “Pathogenic-non-Pathogenic genera” (P-nP(gn)), respectively. Among *M*. *hyopneumoniae* strains, two DR repeats types, 12 SSR and 39 SSRM could be analysed. Higher conservation could be observed in P-nP(sp), and a reversed scenario was seen in P-nP(gn), where nonconserved and absence of tandem repeats were found (Fig 5; S3 Table). These results demonstrated that although the number of DR repeats is almost the same in the orthologous genes of *M*. *hyopneumoniae* strains, the number of SSR and SSRM decreases. Moreover, the numbers of orthologues that share repeat conservation among the pathogenic *M*. *hyopneumoniae* strains and *M*. *flocculare* orthologues drastically decrease (see Fig 5).

In summary, a comparison of *M*. *hyopneumoniae* strain 7448 orthologous genes revealed that 58% of repeats were conserved, 36% were nonconserved and 6% were absent in *M*. *hyopneumoniae* strain 7422 orthologous. Considering *M*. *hyopneumoniae* strain J, the values change to 46% of repeat conservation, 46% of nonconservation and 8% of absence in the orthologous genes. *M*. *flocculare* orthologues demonstrated only 6% of repeat conservation, 44% of nonconservation and 50% of absence. The comparison among the orthologous genes relating to pathogenicity P-nP(sp) showed 40% of repeat conservation and 9% and 6% of nonconservation and absence, respectively. The P-nP(gn) analysis revealed that only 6% of repeats were conserved and 56% were nonconserved or absent among orthologous genes (Fig 5D; S3 Table).

Repetitive elements in the upstream regions of the putative adhesin-coding genes from the three *M*. *hyopneumoniae* strains and *M*. *flocculare* were searched using the same approach as described above. Sixteen *M*. *hyopneumoniae* putative adhesin-coding genes [44] were selected for comparison analysis. In these genes, palindrome repeats (PAL and PALG) were found with an average of 5 repeat elements per gene in *M*. *hyopneumoniae* strain 7448. In the majority of the genes coding for adhesins (12 genes), palindromic elements were located directly in the CDS 5’ upstream region. However, this situation was not applied to MHP7448_0005, MHP7448_0006, MHP7448_0361 and MHP7448_0362 CDSs. Therefore, a search for repetitive elements was performed in the upstream region of Transcriptional Unit (TU)_01 (containing MHP7448_0005 and MHP7448_0006) and TU_63 (containing MHP7448_361 and MHP7448_362). Using this approach, PAL and PALG elements could be found. Comparative analysis revealed that all *M*. *hyopneumoniae* strain 7422 and *M*. *hyopneumoniae* strain J adhesin orthologous genes were present in the respective genome and a palindromic repeat conservation level of 87% and 69%, respectively, were established. Comparison between *M*. *hyopneumoniae* strain 7448 and *M*. *flocculare* demonstrated only 27% of conserved elements in 5’ upstream region of the orthologue genes (Table 2; S4 Table).

**Table 2:** The presence of repetitive elements in upstream regions of putative adhesin-coding genes.

MHP_7448		N° ortholog element /N° MHP_7448 elements		
Gene ID	Product	MHP_7422	MHP_J	MFL
MHP7448_361	P29	5/5 (100%)	5/5 (100%)	3/5 (60%)
MHP7448_362	P69			
MHP7448_497	P76	5/5 (100%)	3/5 (60%)	1/5 (20%)
MHP7448_198	P97	6/6 (100%)	3/6 (50%)	-
MHP7448_108	P97	3/3 (100%)	2/3 (67%)	1/3 (33%)
MHP7448_272	P97	5/6 (83%)	5/6 (83%)	4/6 (67%)
MHP7448_199	P102	4/5 (80%)	5/5 (100%)	1/5 (20%)
MHP7448_107	P102	1/1 (100%)	1/1 (100%)	0/1 (0%)
MHP7448_271	P102	1/1 (100%)	0/1 (0%)	0/1 (0%)
MHP7448_105	P102	3/3 (100%)	1/3 (33%)	3/3 (100%)
MHP7448_663	P146	7/7 (100%)	6/7 (86%)	4/7 (57%)
MHP7448_496	P216	2/2 (100%)	1/2 (50%)	1/2 (50%)
MHP7448_373	LppS	1/2 (50%)	0/2 (0%)	1/2 (50%)
MHP7448_372	LppT	1/5 (20%)	2/5 (40%)	2/5 (40%)
MHP7448_006	MgPa	5/5(100%)	5/5(100%)	3/5(60%)
MHP7448_005	Mgpa			
**Total**		**49/56 (87%)**	**39/56 (69%)**	**24/56 (27%)**

No MHP7448_198 orthologous gene was found in *M*. *flocculare* (MFL) genome using BLAST. Abbreviations: *M*. *hyopneumoniae* strain 7448 (MHP_7448), *M*. *hyopneumoniae* strain 7422(MHP_7422), and *M*. *hyopneumoniae* strain J (MHP_J).

### Experimental validation

Based on *in silico* comparison analysis, 12 CDSs with different tandem repeat compositions in 5’ upstream regions were selected to perform the experimental procedures (3 CDSs with DR repeats, 4 with SSR repeats, and 5 with SSRM repeats). Some of these CDSs encode important proteins to bacterial survival, like transporters or signalling molecules, and others encode hypothetical proteins. Nine genes with palindromic sequences at the upstream region of putative adhesins proteins were also selected (Table 3).

**Table 3:** Influence of repeat composition on mycoplasma gene expression.

MHP_7448			MHP_J			MFL		
Repeat Analyzed	Gene ID	Product	Conservation	Expression	p-value	Conservation	Expression	p-value
DR_01_TE	rpsP	30S ribosomal protein S16	C	up	***	A	-	ns
DR_01_T	MHP7448_0397	hypothetical protein	C	up	**	A	-	ns
DR_06_E	MHP7448_0197	hypothetical protein	NC	-	ns	A	-	ns
SSR_49_LI	MHP7448_0485	hypothetical protein	NC	-	ns	NC	up	**
SSR_30_LI	MHP7448_0484	hypothetical protein	NC	-	ns	NC	up	***
SSR_42_LI	MHP7448_0623	ABC transporter ATP-binding—Pr1	C	-	ns	C	-	ns
SSR_05_LI	MHP7448_0087	GTP-binding protein	A	up	****	A	up	*
SSRM_07_TL	sipS	signal peptidase I	NC	do	***	NC	do	****
SSRM_69_L	glyA	glycine hydroxymethyltransferase	NC	up	*	NC	up	*
SSRM_15_TL	MHP7448_0272	P97-like	NC	up	****	NC	up	***
SSRM_10_L	MHP7448_0108	P97-like copy 2	NC	-	ns	NC	up	*
SSRM_195_L	MHP7448_0505	lipoprotein	C	-	ns	NC	up	***
PALG_E_472^a^								
PALG_E_473^a^								
PALG_E_474^a^	MHP7448_0361	P29	100%	up	***	60%	-	ns
PALG_E_975^a^	MHP7448_0362	P69		up	**		-	ns
PALG_ES_134^a^								
PALG_E_1055	MHP7448_0497	P76	60%	up	***	20%	-	ns
PALG_E_1056								
PALG_E_572								
PALG_E_573								
PALG_ES_183								
PALG_E_291	MHP7448_0108	P97	67%	-	ns	33%	up	*
PALG_E_859								
PALG_S_1184								
PALG_E_381	MHP7448_0272	P97	83%	up	****	67%	up	***
PALG_E_382								
PALG_E_383								
PALG_E_385								
PALG_E_925								
PALG_E_926								
PALG_E_863	MHP7448_0107	P102	100%	NT	NT	0%	up	*
PALG_E_924	MHP7448_0271	P102	0%	up	**	0%	up	**
PALG_E_237^b^									
PALG_E_238^b^									
PALG_E_239^b^	MHP7448_0006	MgPa	100%	-	ns	60%	up	***
PALG_E_240^b^	MHP7448_0005	Mgpa		-	ns		up	*
PAL_ES_24^b^								

Asterisks indicate statistically significant differences in levels of expression, *0.01 < *P* < 0.05, **0.001 < *P* <0.01***, *P* < 0.001****, ns: non-significant. Genes with DNA repeats in the respective upstream region were classified as Conserved (C), Nonconserved (NC) and Absent (A). These genes were analysed and changes in the expression level of *M*. *hyopneumoniae* strain J (MHP_J) and *M*. *flocculare* (MFL) in relation to *M*. *hyopneumoniae* strain 7448 (MHP_7448) through qPCR assays were evaluated. Upregulation (up), downregulation (do) or no significantly differences in expression (-) could be observed among tested genes.

^(a)^ PALG elements found in the 5’ upstream region of the first gene of the transcriptional unit containing MHP7448_0361 and MHP7448_0362 CDS.

^(b)^ PALG elements found in the 5’ upstream region of the first gene of the transcriptional unit containing MHP7448_0005 and MHP7448_0006 CDS.

NT = not tested

Comparative transcription analysis were performed considering the conservation level of SSR, SSRM and DR elements in upstream regions of genes from *M*. *hyopneumoniae* strain J and *M*. *flocculare*, which are orthologous to *M*. *hyopneumoniae* strain 7448. Results revealed that, when conserved repeat (SSR_42_LI in both mycoplasmas analysed and SSRM_195_L in *M*. *hyopneumoniae* strain J), were found in upstream region of the gene, nonsignificant differences in the basal transcription level were observed. In the same way, in genes with nonconserved SSR repeats in intergenic region (SSR_49_LI and SSR_30_LI in *M*. *hyopneumoniae* strain J) nonsignificant differences in gene expression were seen. However, when nonconserved SSR repeats were analysed in *M*. *flocculare*, differences in gene expression were observed. The absence of SSR element (SSR_05_LI) in *M*. *hyopneumoniae* strain J and *M*. *flocculare* upstream region of orthologous demonstrated different transcription profile (Table 3). Genes with nonconserved SSRM elements (SSRM_07_TL, SSRM_69_L and SSRM_15_TL) in upstream region of *M*. *hyopneumoniae* strain J and M. flocculare demonstrated changes in level of transcripts. The exception was SSRM_10_L which only *M*. *flocculare* showed differences in gene expression (Table 3). Interestingly, genes with DR repeats in the upstream region displayed a different expression profile in relation to those with SSR and SSRM repeats, as only conserved elements (DR_01_TE; DR_01_T) demonstrated distinct expression profiles between *M*. *hyopneumoniae* strain 7448 and J (Table 3).

Detailed analyses of conserved SSR elements found in the 5’ upstream region of MHP7448_0623 orthologues from *M*. *hyopneumoniae* strain J and *M*. *flocculare* showed no significant differences in basal gene transcription levels in relation to *M*. *hyopneumoniae* strain 7448 (S1 Fig). However, among the mycoplasma orthologues, the *glyA* gene displays a nonconserved SSRM in the 5’ upstream region and exhibited differential expression in the tested condition (S1 Fig). The SSR element absence in MHP7448_0087 orthologues from *M*. *hyopneumoniae* strain J and *M*. *flocculare* also seems to influence in transcriptional, whereas differences in gene expressions were observed (S1 Fig).

As demonstrated in tandem repeats, adhesins coding genes revealed similar profile when conservation of palindromic elements was related to transcriptional levels. Conserved (100%) PAL and PALG present in MHP7448_0005 and MHP7448_0006 orthologues from *M*. *hyopneumoniae* strain J did not affect gene expression. However, when relative conservation dropped to 60% in orthologues of *M*. *flocculare*, basal transcript level varied significantly. The absence of palindromic repeats in MHP7448_0271 orthologues from *M*. *hyopneumoniae* strain J and *M*. *flocculare* resulted in significant differences in basal gene transcription level. Furthermore, even when few elements were lost in MHP7448_0272 orthologues from *M*. *hyopneumoniae* strain J and *M*. *flocculare*, distinct transcripts level were observed (Table 3 and S2 Fig). A detailed comparison of palindromic elements in adhesin-coding genes that were experimentally analysed is described in S4 Table.

In summary, the presence of nonconserved repetitive DNA elements (SSR and SSRM) among the 5’ upstream regions of *M*. *flocculare* orthologous had gene expression variations under the tested conditions. In *M*. *hyopneumoniae* strain J only the classified absent repeats (SSR) demonstrate some potential bias in transcription. In relation to PAL and PALG the loss of some elements (lower conservation) could be related to the observed differences in gene expression. Orthologous of MHP7448_497, MHP7448_272 and MHP7448_272 in *M*. *hyopneumoniae* strain J and MHP7448_108, MHP7448_107, MHP7448_271 and MHP7448_272 could support this data (Table 3). All these findings suggest a putative regulatory influence on gene expression when tandem or palindromic repeats were present in 5’ upstream region of the gene.

### Repeat presence in differentially regulated CDS

In order to understand the possible role of repetitive elements in transcriptional regulation, a search for the presence of palindromic elements was performed in genes with differential expression profiles, previously reported [46–50]. Since the molecular pathways that could modulate the regulation of these genes are unknown, the presence of conserved palindromic repeats in intergenic region could suggest a probably source of cis-regulatory elements. A total of 243 differentially expressed genes in *M*. *hyopneumoniae* strain 232 were compared against the *M*. *hyopneumoniae* strain 7448 genome (S5 and S6 Tables). Orthologous could be assigned (using BLAST approach) to 206 out of 243 (85%) of the differentially expressed genes, in the *M*. *hyopneumoniae* strain 7448 genome. An average of 4 palindromic elements was present in intergenic regions of these 206 orthologous genes present in *M*. *hyopneumoniae* strain 7448 (S6 Table). Comparison with orthologous genes from *M*. *hyopneumoniae* strain 232 showed that 103 (61%) genes had exactly the same element found in *M*. *hyopneumoniae* strain 7448 and only 17 (10%) did not have any corresponding elements (S5 and S6 Tables). In conclusion, almost 80% of the palindromic elements were conserved among the differentially expressed genes analysed. The ΔG values of PAL and PALG repeats found were evaluated and in general, demonstrated satisfactory potential to form secondary structures (S5 and S6 Tables).

## Discussion

Prokaryote genomes are extremely diverse in terms of nucleotide composition and the presence of distinct patterns of repeat sequences that could affect the physical properties of DNA molecules [36]. In this work, a global analysis of tandem and palindromic repetitive elements found in noncoding sequences of *M*. *hyopneumoniae* strain 7448 was reported. *In silico* analysis revealed that the majority of the repeat sequences found were classified as palindromic elements (1,171 elements) compared with tandem repeats (144 elements). Similar results were also reported by Huang et al. [36] who investigated tandem and palindromic repeats in protein-coding sequences and intergenic regions through a global analysis of more than 1,000 genomes, including *Mycoplasma agalactiae*, *M*. *bovis*, *M*. *fermentans* and *M*. *mycoides*. Further analysis of the repetitive elements identified in our work demonstrated that common motifs between tandem or palindromic repetitive elements could not be established. The pattern frequently observed was the presence of AT-rich sequences in all elements investigated. In SSRM, for example, mononucleotides containing only Adenine (A) or Thymine (T) repeated 8 to 25 times were observed, in accordance with the AT-rich genome of *M*. *hyopneumoniae* strain 7448 [1].

Combinations of palindromic elements, tandem repeats and promoter sequences could be detected in 92% of all *M*. *hyopneumoniae* strain 7448 transcriptional units demonstrating that different mechanisms of regulation can be considered (see S2 Table). Results showed that 83% of SSR and SSRM repeats were located proximal to putative promoter sequences and 76% of palindromic elements revealed similar profile. The totality of tandem repeat class was associated with promoters (S2 Table). Previous work demonstrated that copy number of tandem repeats next to putative promoter sequences can modulate RNA polymerase action, by spacing the promoter region in distinct way, affecting gene expression [54]. Moreover, palindromes can form cruciform structures and mediate promoter sequence availability or create physical barriers that could be broken in a regulatory way [53]. DNA repeats have already been described as being involved in phase variation resulting in diversity of pathogenic phenotypes and other bacterial biological processes [19]. Therefore, aiming to establish the role of the repetitive elements found in *M*. *hyopneumoniae* strain 7448, a comparative investigation of tandem elements was performed in relation to mycoplasma genomes. Although subtle differences were observed, in general, the level of repeat conservation was higher between the two pathogenic *M*. *hyopneumoniae* strains (7448 and 7422), compared with nonpathogenic mycoplasmas (*M*. *hyopneumoniae* strain 7448 versus *M*. *hyopneumoniae* strain J and *M*. *hyopneumoniae* strain 7448 versus *M*. *flocculare*) as shown in Fig 5. Adhesin proteins are known to be essential to mycoplasma host infection and for the establishment of the disease [44]. To evaluate the correlation of the palindromic repeats associated with these genes, another comparison analysis was done. A comparative analysis of the PAL and PALG elements among the orthologue adhesin genes in the three *M*. *hyopneumoniae* strains and *M*. *flocculare* was performed with similar results as previous demonstrated for the tandem repeats. Adhesin-coding genes show greater differences in the number of palindromes in non-pathogenic strains (*M*. *hyopneumoniae* strain J and *M*. *flocculare*) than the two *M*. *hyopneumoniae* pathogenic strains (7448 and 7422). An example was seen in MHP7448_0107, MHP7448_0271 and MHP7448_497 genes, which showed 100% conservation in *M*. *hyopneumoniae* strain 7422 and a marked reduction in palindromic elements in *M*. *hyopneumoniae* strain J and *M*. *flocculare*. In this work tandem repeats (SSR and SSRM) were also reported in 3 of the 16 putative adhesin coding genes analysed (MHP7448_0108, MHP7448_0272 and MHP7448_0373). Previous authors have reported that tandem repeats, more precisely SSR, were able to influence adhesin gene expression [21, 25] but differences in palindromic repeats had not yet been reported.

To know the role of the repetitive elements found among the 5’ upstream regions in different CDSs, an experimental analysis was performed with orthologous genes from two *M*. *hyopneumoniae* strains (7448 and J) and *M*. *flocculare*. Detailed analysis of comparative studies of the presence of tandem repeats (SSR, SSRM and DR) in different genes and palindromic elements found in putative adhesin-coding genes were the basis of the experimental investigation. In general, the results of qPCR assay revealed that conserved elements among distinct mycoplasmas seem to have no influence on gene expression, as observed in the MHP7448_0623 gene (Table 3). Whereas when nonconserved or absent elements were investigated, a relation between differences in repetitive elements found in 5’ upstream regions and gene expression could be suggested (Table 3). Interestingly, a SSR found in the 5’ upstream region of the MHP7448_0087 (GTP-binding protein) gene was conserved in all pathogenic strains of mycoplasma (7448 and 7422) and absent in nonpathogenic mycoplasmas (*M*. *hyopneumoniae* strain J and *M*. *flocculare*). The same pattern was present in SSRM found in the 5’ upstream region of the *glyA* (glycine hydroxymethyltransferase) and *sipS* (signal peptidase I) genes, which are conserved in pathogenic mycoplasmas strains and nonconserved in nonpathogenic mycoplasmas (S3 Table). Differences in gene expression were observed among tested mycoplasma RNAs of *M*. *hyopneumoniae* strain J and *M*. *flocculare* in relation to *M*. *hyopneumoniae* strain 7448 in MHP7448_0087, *glyA* and *sipS* genes (Table 3), suggesting that some DNA repeats analysed may be involved in mycoplasma pathogenicity. GTP-binding protein, glycine hydroxymethyltransferase enzyme and signal peptidase I coding genes are important to cell viability and in some cases have already been related to mycoplasma pathogenicity process [55–57]. Promoter sequences associated with tandem repeats were present in all regulatory sequences of experimentally tested genes (S2 Table), reinforcing the hypothesis that repetitive elements may be interfering in the promoter region and consequently the transcription of related genes [20, 54].

The adhesin-coding genes are important virulence factors and seem to be regulated depending on the presence or absence of palindromic repeats. Therefore, it is possible to suggest a relation between the differences in gene transcriptional level and the divergence of DNA repeats found in the 5’ upstream regions among mycoplasma orthologous genes, as observed in genes coding for adhesin proteins P102 (MHP7448_0271) and P97 (MHP7448_0272) and genes coding for proteins in the MgPa operon (MHP7448_0005 and MHP7448_0006). Similar results were previously demonstrated for genes coding adhesin in *M*. *genitalium* (MgPa adhesin proteins) [21] and in *M*. *gallisepticum* (M9/pMGA adhesin proteins) [25], whereas repetitive DNA could influence transcriptional regulation.

The repeats found in the *M*. *genitalium* genome represent hypervariable sites in the MgPa operon, which encodes two adhesin proteins that represent surface proteins required for the development of the terminal organelle structure and attachment of the organism to host epithelial cells. Such adhesin variation may allow this organism to evade the host immune response and to adapt to diverse host microenvironments, thus establishing persistent infection [21]. In *M*. *gallisepticum*, the GAA trinucleotide repeat region regulates M9/pMGA gene expression (which encodes adhesin(s) associated with haemagglutination). Depending on the copy number of GAA in the intergenic regions, gene expression can be inhibited [25]. In *M*. *bovis*, repetitive elements were involved in a family of phase- and size-variable membrane surface lipoprotein antigens [27].

Besides transcriptional regulation, DNA repeats could also be involved in translation regulation. Identification of transcription start site (TSS) was performed for 23 genes of *M*. *hyopneumoniae* strain 7448 [12]. The analysis of these 23 genes in relation to the presence of DNA repeats revealed that 15 of them have at least one repetitive element located in the 5’ UTR region (S7 Table). This data demonstrates another potential mechanism of regulation by DNA repeats as they could influence in the process of mRNA translation into its protein product.

Previous studies have demonstrated differential expression of genes from *M*. *hyopneumoniae* 232 growing under several specific culture conditions [46–50], so a detailed analysis was performed to better understand the differences in transcriptional level of genes that diverge in the presence of repetitive elements in the 5’ upstream region. All *M*. *hyopneumoniae* strain 7448 orthologues from differentially expressed genes of the *M*. *hyopneumoniae* strain 232 were selected and analysed for repeat conservation. This study revealed that almost all differentially expressed genes had palindromic elements in their 5’ upstream regions. Orthologous gene comparisons between the two *M*. *hyopneumoniae* strains (7448 and 232) showed that the PAL and PALG repeats were approximately 80% conserved in *M*. *hyopneumoniae* strain 232, reinforcing the putative regulatory role of these repetitive elements since temperature, pH and others factors can influence the formation of secondary structures, as well as could perturb the stability of the nucleotide bindings [58].

In this work, the presence of SSR, SSRM, DR, PAL and PALG DNA repeats found in the 5’ upstream regions of *M*. *hyopneumoniae* strain 7448 CDSs was described. Relevance in transcriptional regulation of the DNA repeats found could be established through comparison analysis, demonstrating that repeats could be perpetuated among related mycoplasmas, and some of them could be involved in pathogenicity. Experimental assays revealed differential expression of genes differing in repetitive elements in the 5’ upstream region, reinforcing the putative regulatory role of palindromic and tandem repeats. Previously described differentially expressed genes show palindromic elements in the upstream region of the start codon and are conserved between two strains of *M*. *hyopneumoniae*. All of these findings suggest the importance of repetitive DNA elements in *M*. *hyopneumoniae*, and contribute to expand the source of regulatory sequences that can modulate gene expression in the important pig pathogen *M*. *hyopneumoniae*.

## Supporting Information

S1 Fig Experimental validation through qPCR assay of tandem elements comparison. (PDF)

S2 Fig Experimental validation through qPCR assay of palindrome elements comparison in adhesin coding genes. (PDF)

S1 Table Primers used in qPCR assays. (PDF)

S2 Table DNA repeats after filtering parameters. (XLSX)

S3 Table Tandem comparison among related mycoplasmas. (XLSX)

S4 Table Palindrome elements in adhesins coding genes comparison among related mycoplasmas. (XLSX)

S5 Table Presence of repeat elements on upstream regions of regulated genes in *M*. *hyopneumoniae* strain 232 (PDF)

S6 Table Palindrome elements comparison analysis in regulated genes of *M*. *hyopneumoniae* strain 232 (XLSX)

S7 Table DNA repeats potentially associated with translational regulation. (PDF)
