# Supplementary material for: Repetitive Elements in Mycoplasma hyopneumoniae Transcriptional Regulation
Source: PLoS One. 2016 Dec 22;11(12):e0168626. doi: 10.1371/journal.pone.0168626 (PMC5179023; doi:10.1371/journal.pone.0168626)
Supplement: S1 Fig — (PDF) [file pone.0168626.s001.pdf]

Supplementary Fig. S1: Experimental validation through qPCR assay of tandem elements comparison.

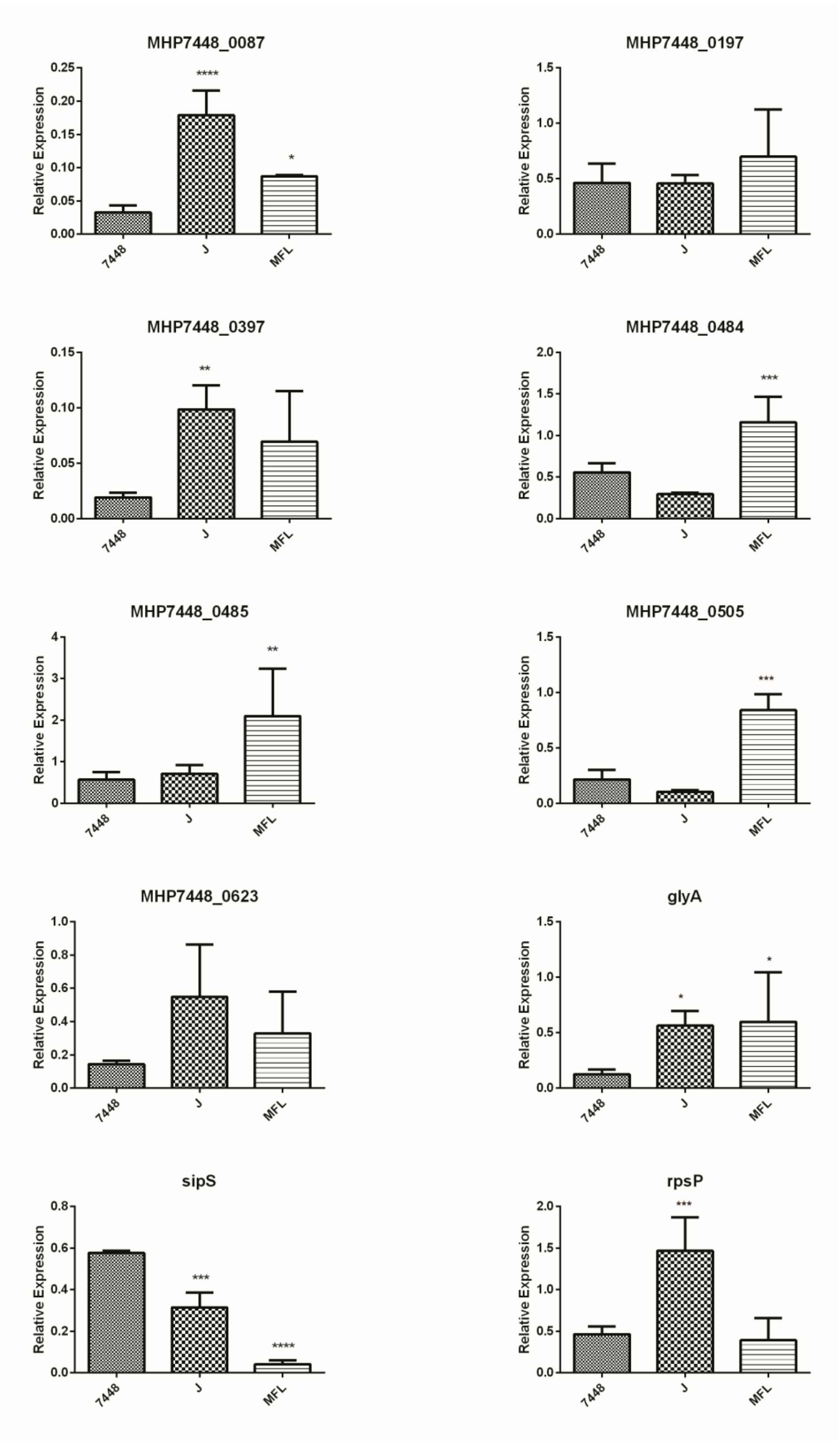

Asterisks indicate statistically significant differences in levels of expression, *0.01 < P < 0.05, **0.001 < P <0.01, ***P < 0.001. Abrevitions: *M. hyopneumoniae* strain 7448 (7448); *M. hyopneumoniae* strain J (J) and *M. flocculare*.
