# Supplementary material for: Repetitive Elements in Mycoplasma hyopneumoniae Transcriptional Regulation
Source: PLoS One. 2016 Dec 22;11(12):e0168626. doi: 10.1371/journal.pone.0168626 (PMC5179023; doi:10.1371/journal.pone.0168626)
Supplement: S2 Fig — (PDF) [file pone.0168626.s002.pdf]

Supplementary Figure S2: Experimental validation through qPCR assay of palindrome elements comparison in adhesin coding genes.

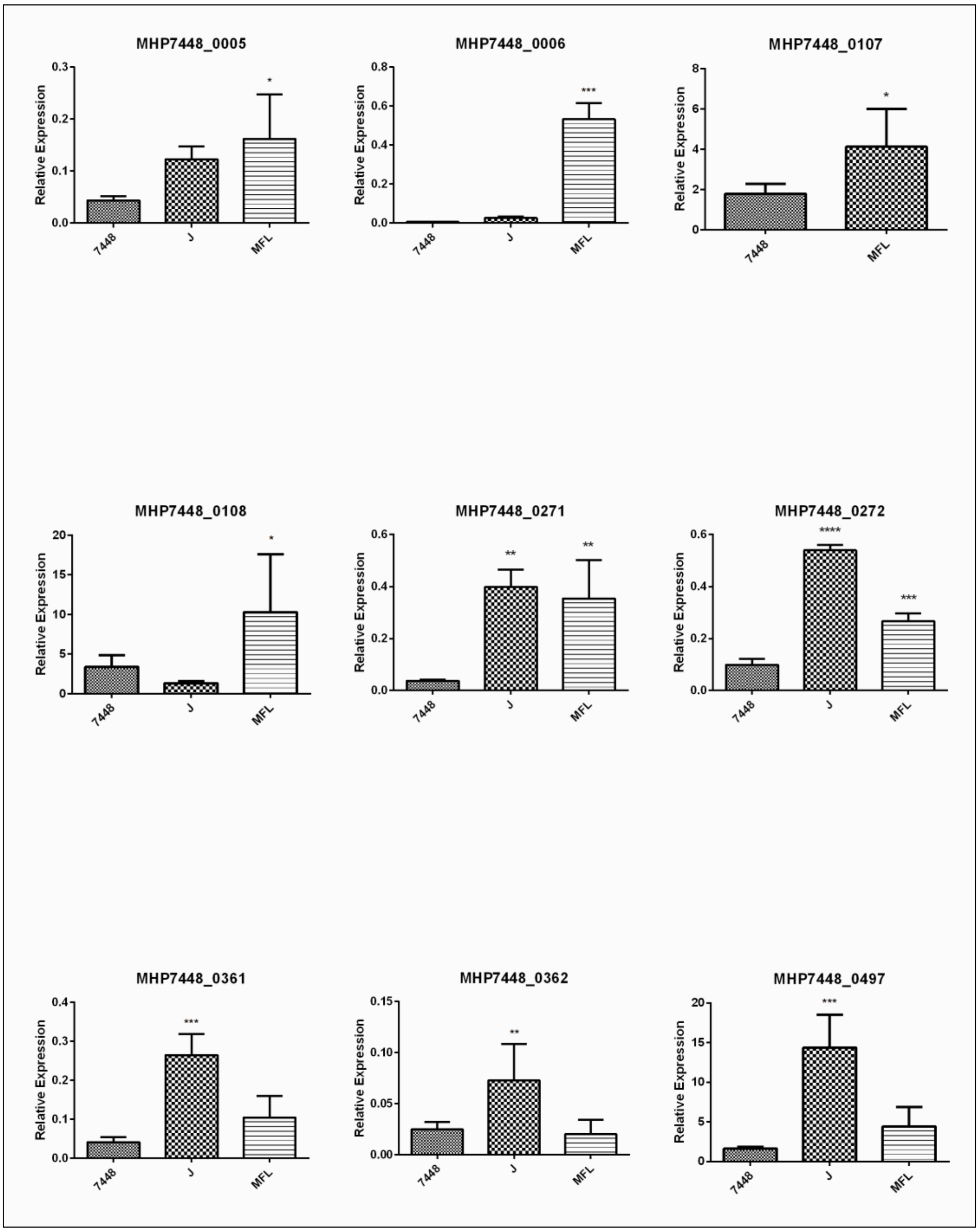

Asterisks indicate statistically significant differences in levels of expression, *0.01 < P < 0.05, **0.001 < P <0.01, ***P < 0.001. Abbreviations: *M. hyopneumoniae* strain 7448 (7448); *M. hyopneumoniae* strain J (J) and *M. flocculare* (MFL).
