# Supplementary material for: Repetitive Elements in Mycoplasma hyopneumoniae Transcriptional Regulation
Source: PLoS One. 2016 Dec 22;11(12):e0168626. doi: 10.1371/journal.pone.0168626 (PMC5179023; doi:10.1371/journal.pone.0168626)
Supplement: S1 Table — (PDF) [file pone.0168626.s003.pdf]

Supplementary Table S1: Primers used in qPCR assays

Target (gene_name)	Primer_name	Sequence (5´- 3´)	Lenght (bp)	TM (°C)	Product (bp)	Analysis
MYF_00025	005 _MFL_F	GATCCAACTTTTTCTGCCACTTCA	24	56.0	156	A
	005 _MFL_R	CGCAAATGTTCTTGACGACGTTG	23	57.3		
MHP7448_0005	005 F	GAACAGAACAAGAAGTCGCTTTTT	24	54.3	160	A
	005 R	GCATCGCCAATGTTTGCCAAA	21	57.6		
MYF_00030	006 _MFL_F	CCTTCAAATTCTGTACGCCCCAAT	24	57.7	162	A
	006 _MFL_R	TAAATGACCGCCACCGCCTCA	21	61.4		
MHP7448_0006	006 F	GACCAGACGGGGATTGTATTGG	22	57.6	184	A
	006 R	GCATCAACAATTATCGCCAAAGAA	24	54.5		
MYF_00550	107 _MFL_F	GATGCAAAGCCAACAAATCCGAGC	24	59.5	172	A
	107 _MFL_R	GCGTCTCTTTTGGACTTGCTGGA	23	59.7		
MHP7448_0107	107 F	AGCTTTATTCACTACCTTCAGCAC	24	55.0	165	A
	107 R	GTTGTTGGGCTGGCTTCTGAC	21	59.0		
MHP7448_0108	108 F	CGGCGTCAGTTCTGCCAAA	19	58.6	173	A/T
	108 R	CTCCGATTTTGCTCCATCTCGA	22	56.9		
MHJ_0105	108_J F	AATCAGAAACAGCTAAACCCGTGG	24	57.6	155	A/T
	108_J R	CTCCGATTTTGCTCCATCACGA	22	57.4		
MYF_00575	108_MFL F	TGAAAGCGGAATTGGATTTTATGG	24	54.2	168	A/T
	108_MFL R	GAGACTGCTTGTTGTGACCCG	21	58.3		
MYF_00545	197_MFL F	GCAATCTTTCGGCGTAAAATACAG	24	54.8	175	T
	197_MFL R	TTAAGGCGGTGGCAATCG	18	55.8		
MHP7448_0197	197F	TCGGAGAAAATTACAAAAACGCTC	24	54.1	168	T
	197R	CTAAGAACGCTGCTAAGGGCAG	22	57.9		
MYF_01800	271 _MFL_F	TCACAGTAGTTTTGATGTTGCAGC	24	56.0	167	A
	271 _MFL_R	CTTGGGTCACTGTATTTCTTTGCG	24	56.4		
MHP7448_0271	271 F	CATTTTCACGGCTTGATTCAGGGC	24	59.1	174	A
	271 R	GTCCTGCAATTCCTTGGATTGTGG	24	58.5		
MHP7448_0272	272 F	TCTAACTTGATTTTACGAGCACGC	24	55.5	163	A/T
	272 R	GCTCAGTTAATTTTGCCTCAAGAT	24	54.0		
MYF_01805	272_MFL F	GAAAATTAGCGGGTTTGGGCA	21	56.4	182	A/T
	272_MFL R	TTTCTGCAACAATAAGTTGTCCA	23	53.2		
rpsP	286 F	CAACCCGTTCTACAAAATTGTCGT	24	56.0	163	T
	286 R	AACCGTTTGAGTTACTTGTGCG	22	56.0		
MF01456	286_MFL_F	CGACTTCAAAGAAAGGGTTCGA	22	55.2	191	T
	286_MFL_R	CTAACAGTGTTTGTCGATTGAGCG	24	56.3		
MYF_01300	361 _MFL_F	CCAAGCGGGGTAGGAAAAACATC	23	58.4	159	A
	361 _MFL_R	AGTTGGAATCTGGCTTAAAAACCC	24	55.8		
MHP7448_0361	361 F	CCAAGTGGTCAGGGCAAATCATC	23	58.4	159	A
	361 R	AGTAGCCGTCTGACTTAAAAATCC	24	54.8		
MYF_01305	362 _MFL_F	TGTTTCGATAATTTCGCTTGCTTC	24	54.4	180	A
	362 _MFL_R	ATTGCCGCTCATAAAATTGGGC	22	56.8		
MHP7448_0362	362 F	TACGATTAAATACACGCTTGCTGG	24	55.4	168	A
	362 R	CGCATCGGGTCGATTACAAAA	21	55.7		
MHP7448_0397	397 F	CCGCTGACTCTGACATCCTTTTGC	24	60.2	179	T
	397 R	GCACCAGAGGAACCAAACGGA	21	59.9		
MF00009	397_MFL_F	CAAAATAGCACTTCCAAAACGATT	24	52.6	174	T
	397_MFL_R	GGTATAATCCCATATTCCCAAGTT	24	52.3		
MHP7448_0484	484 F	GTCTCTGGCTCTGAATTGGTCGG	23	59.5	188	T
	484 R	CGGGTTACCATAACTTACAGTCGC	24	57.4		
MYF_00935	484_MFL F	TTCTGTGAATCTGAATTGGGCGA	23	56.7	160	T
	484_MFL R	CCATCATCCTCTGAACCTGAAAAA	24	55.1		
MHP7448_0485	485 F	ACGGTTCAATTTCCCTTAAAGAGC	24	55.7	158	T
	485 R	CTTTTTCCCCTTGAAGATGTGGG	23	56.2		
MF00865	485_MFL F	GGCAATCCGTTAAGCTCACG	20	56.5	165	T
	485_MFL R	GGCAATTTCAAAGACGAATTTTTG	24	52.3		
MYF_00885	497 _MFL_F	CGGTAGTGATGCCCAACAACAAA	23	58.0	163	A
	497 _MFL_R	CCCCTCAGTATTGTGCTGTTCAG	23	57.6		

MHP7448_0497	497 F	GATCCAGTGGTGGAAGTTCCTCAG	24	59.0	175	A
	497 R	GCTGGCTTTTCTGCTTCTTTAGGC	24	58.8		
MHP7448_0505	505 F	CCGAATTTTTTGCTACTTCGCTTG	24	55.4	166	T
	505 R	CGCTCACTTGGAACCGAAAGTC	22	58.4		
MF00046	505_MFL F	GCGAACAGACAATTATGGGTGTT	23	55.9	157	T
	505_MFL R	GAGCCAGGCACAAGAACTGAATAG	24	57.8		
MHP7448_0525	525 F	CCAAAAATGATTCCAGGGAA	20	56.3	190	GR
	525 R	GGCGTTTTAATTACACCGG	19	58.0		
MYF_00795	525_MFL_F	GCCGCTTTTTCAAAATCTTGGTTT	21	55.5	160	GR
	525_MFL_R	GATATCAATTCCACCAAAACGGTT	24	53.9		
MHP7448_0623	623 F	TCGAAGACACTTTGGCAAACA	21	55.1	165	T
	623 R	CCAATGCAACTTTTTGTTTTTCGC	24	54.9		
MYF_02820	623_MFL F	GTGAAAAACAGAAAATTGCACTTG	24	52.2	171	T
	623_MFL R	CTTCTGGAATATGATGGCTTACAA	24	52.9		
MHP7448_0081	81 F	CGAGACAAAAAGGTTCAAAAAG	22	57.1	171	GR
	81 R	GTAAAAAGGGATGTGCCTGC	20	60.4		
MF01193	81_MFL_F	GGGCTGATTTTAACGGGTGCTT	22	58.2	162	GR
	81_MFL_R	CGGAGCAACGCTTTGAAAAGTCTC	24	58.8		
MHP7448_0087	87 F	GTTGTCGGAGTGCCTAACACTGG	23	59.9	154	T
	87 R	GCAATAGGATACCTGGGGTGTC	22	57.2		
MF01266	87_MFL F	CGGGAATTTTGCCACCTAAA	20	53.5	183	T
	87_MFL R	CAGCCTCATTTTCAGCAGGTTT	22	56.1		
deoC	deoC F	GCTTTGATTGCCAATTTTTCG	21	56.7	163	GR
	deoC R	CAGCCCTTTTATCACCTCATG	21	60.6		
deoC	deoC_MFL_F	GCCAGTTTGGGTTAGGCTTG	20	56.7	160	GR
	deoC_MFL_R	CATTACCATGTCAATTTCGTCAGC	24	54.7		
glyA	glyA_MFL F	GTGGCTATTCTGCTTATTCAGGGA	24	56.7	168	T
	glyA_MFL R	GGGTTGTGGCGGTTATCACATG	22	58.7		
glyA	glyAF	TTGATTTTGCCCGCTTTCGAC	21	56.7	170	T
	glyAR	GCCACCTCTTGGTCCTCTCAAAG	23	59.6		
lon	lon F	TAAAACTTCAAGCATCGCCG	20	58.4	165	GR
	lon R	GAAATCCGAGGACATCGCC	19	62.3		
lon	lon_MFL_F	CGGGCGTGGGGATATTAAACTAAC	24	57.6	176	GR
	lon_MFL_R	AAGGTCCATCTTTTGGAATTGCC	23	56.0		
sipS	sipS F	CGCTCACTTGGAACCGAAAGTC	22	55.6	179	T
	sipS R	CCTTCGGGAATAACTCCGTTAA	22	54.4		
MYF_01338	sipS_MFL F	GTGGTTTTCAAATATAAGGAGCAA	24	51.8	167	T
	sipS_MFL R	AATTTACCTTCAGGAATTGTGCC	23	53.8		
upp	upp F	GGCTCTAAAACAATCGCAACTG	22	60.8	153	GR
	upp R	CGGTTTGGGTATGCTTGACTC	21	62.6		
upp	upp_MFL_F	CAACACAAGTTGTTTTTACCCCG	23	55.1	173	GR
	upp_MFL_R	GGCAACAGAATCGGCATCAGG	21	58.8		

Primers were used in the follow analysis: Adhesins coding genes (A), Tandem repeats (T) and establishment of gene reference (GR) used in $2^{-\Delta Ct}$ calculus. Primers used for *M. hyopneumoniae* strain 7448 were the same used for strain J, except for gene MHP7448_0108.
