# Supplementary material for: Repetitive Elements in Mycoplasma hyopneumoniae Transcriptional Regulation
Source: PLoS One. 2016 Dec 22;11(12):e0168626. doi: 10.1371/journal.pone.0168626 (PMC5179023; doi:10.1371/journal.pone.0168626)
Supplement: S5 Table — (PDF) [file pone.0168626.s007.pdf]

Supplementary Table S5: Presence of repeat elements on upstream regions of regulated genes in *M. hyopneumoniae* strain 232

MHP_232		MHP_7448	MHP_7448 x MHP_232	
Gene_name	Product	Gene_name	Conservation[1]	ΔG[2]
mhp004	hypothetical protein	MHP7448_004	5/5 (100%)	-8.06
mhp024	hypothetical protein	MHP7448_022	2/2 (100%)	-2.14
atpB<br>glyS	ATP synthase A chain<br>glycyl-tRNA synthetase	atpB<br>glyS	2/2 (100%)	-5.61
mhp232	hypothetical protein	MHP7448_0685	0/1 (0%)	*
spoU	rRNA methylase	spoU	0/1 (0%)	*
mglA	ATP-binding protein	mglA	2/2 (100%)	-6.32
mhp325	hypothetical protein	MHP7448_0312	4/5 (80%)	-2.61
mhp326	hypothetical protein	MHP7448_0313	5/5 (100%)	-4.34
mhp355	hypothetical protein	MHP7448_0344	4/4 (100%)	-4.85
mhp366	hypothetical protein	MHP7448_0355	2/2 (100%)	-3.33
mhp516	hypothetical protein	MHP7448_0518	1/4 (25%)	-3.13
mhp623	hypothetical protein	MHP7448_0604	4/5 (80%)	-4.18
ftsY	cell division protein	ftsY	2/2 (100%)	-5.09
gatA	glutamyl-tRNA amidotransferase subunit A	gatA	0/5 (0%)	*
mhp115	ABC transporter ATP binding protein	MHP7448_0272	5/7 (71%)	-3.59
rpl4<br>rpl23	50s ribosomal protein L4<br>50s ribosomal protein L23	rplD<br>rplW	6/6 (100%)	-3.82
mhp262	hypothetical protein	MHP7448_0118	4/4 (100%)	-6.09
pfkA	6-phosphofructokinase	pfkA	1/1 (100%)	-2.90
mhp293	hypothetical protein	MHP7448_0331	0/3 (0%)	*
mhp424	hypothetical protein	MHP7448_408	2/2 (100%)	-2.34
mhp451	hypothetical protein	MHP7448_452	3/3 (100%)	-6.91
mhp452	hypothetical protein	MHP7448_453	0/3 (0%)	*
napA	neutrophil activating protein	napA	3/3 (100%)	-4.83
gyrA	DNA gyrase subunit A	gyrA	6/6 (100%)	-3.79
secA	preprotein translocase subunit	MHP7448_0086	4/5 (80%)	-7.33
mhp019	hypothetical protein	MHP7448_017	0/3 (0%)	*
mhp027	hypothetical protein	MHP7448_025	4/4 (100%)	-3.08
mhp324	hypothetical protein	MHP7448_0311	4/4 (100%)	-2.55
aprE	subtilisin-like serine protease	MHP7448_0332	2/4 (50%)	-4.48
mhp354	hypothetical protein	MHP7448_0342	6/7 (86%)	-9.34
mhp360	hypothetical protein	MHP7448_0349	4/4 (100%)	-2.76
gap	glyceraldehyde 3-phosphate dehydrogenase	MHP7448_035	3/3 (100%)	-4.70
mhp630	hypothetical protein	MHP7448_0611	4/4 (100%)	-4.03
rpsG	30s ribosomal protein S7	MHP7448_076	1/1 (100%)	-2.30
rps17	30s ribosomal protein S17	MHP7448_185	6/6 (100%)	-3.82
mhp093	hypothetical protein	MHP7448_287	5/6 (83%)	-7.85
yx1	hypothetical protein	MHP7448_498	5/6 (83%)	-5.62

mhp508	hypothetical protein	MHP7448_511	2/2 (100%)	-0.67
mhp561	hypothetical protein	MHP7448_544	1/4 (25%)	-2.74
mhp662	hypothetical protein	MHP7448_642	3/4 (75%)	-7.42
mhp371	putative high affinity transport system protein p37 precursor	MHP7448_0360		
potA	ABC transporter ATP-binding protein	MHP7448_0369	3/5 (60%)	-0.87
ugpA	sn-glycerol-3-phosphate transport system permease	MHP7448_0370		
fusA	GTP-binding protein chain elongation factor ef-g	fusA	4/5 (80%)	-1.12
trsE	transfer complex protein	MHP7448_0247	5/5 (100%)	-10.43
mhp118	hypothetical protein	MHP7448_0261	2/2 (100%)	-2.99
mhp395	hypothetical protein	MHP7448_0383	1/5 (20%)	-3.59
mhp629	hypothetical protein	MHP7448_0610	3/3 (100%)	-7.07
mhp092	hypothetical protein	MHP7448_340	1/4 (25%)	-2.12
oppD	oligopeptide transport system permease protein	MHP7448_502	5/6 (83%)	-5.62
pgiB	phosphoglucose isomerase B	MHP7448_531	1/2 (50%)	-2.03
pyrH	uridylate kinase	MHP7448_535	2/2 (100%)	-6.29
mhp576	hypothetical protein	MHP7448_556	0/3 (0%)	*
mhp654	hypothetical protein	MHP7448_633	7/7 (100%)	-4.31
p115	p115 protein	MHP7448_657	1/1 (100%)	-3.88
nrdE	ribonucleoside-diphosphate reductase alpha chain	nrdE	2/5 (40%)	-2.23
mhp130	putative DNA processing protein	smf	5/5 (100%)	-10.43
truB	tRNA pseudouridine synthase B	truB	1/1 (100%)	-3.26
mhp170	hypothetical protein	MHP7448_0211	2/3 (67%)	-1.70
oppF	oligopeptide transport system permease protein	oppF-1		
rpl5	50s ribosomal protein L5	MHP7448_0182		
rps8	30s ribosomal protein S8	rpsH	6/6 (100%)	-3.82
adk	adenylate kinase	adk		
mhp247	hypothetical protein	MHP7448_0135	1/1 (100%)	-0.62
mhp273	hypothetical protein	MHP7448_0106	1/3 (33%)	-1.91
mhp303	hypothetical protein	MHP7448_0342	2/7 (29%)	-0.89
gtp1	GTP-binding protein	ychF	2/2 (100%)	-4.04
rps18	30s ribosomal protein S18	rpsR		
mhp517	hypothetical protein	MHP7448_0519	1/1 (100%)	-2.02
mhp309	hypothetical protein	MHP7448_297	0/1 (0%)	*
mhp312	hypothetical protein	MHP7448_299	2/2 (100%)	-3.28
mhp341	hypothetical protein	MHP7448_330	1/1 (100%)	-0.70
mhp362	hypothetical protein	MHP7448_351	3/3 (100%)	-1.39
rluD	ribosomal large subunit pseudouridine synthase D	MHP7448_458	4/4 (100%)	-4.83
mhp472	hypothetical protein	MHP7448_474	4/4 (100%)	-6.68
mhp480	hypothetical protein	MHP7448_482	6/6 (100%)	-3.87
mhp583	hypothetical protein	MHP7448_566	1/4 (25%)	-3.61
mhp634	hypothetical protein	MHP7448_0615	4/4 (100%)	-4.03
rplL	50s ribosomal protein L7/l12	MHP7448_0618		

rpoC	DNA-directed RNA polymerase beta chain	MHP7448_0616	1/1 (100%)	-1.72
cysS	cysteinyl-tRNA synthetase	MHP7448_641	3/4 (75%)	-7.42
mhp670	hypothetical protein	MHP7448_649	1/4 (25%)	-7.32
mhp140	hypothetical protein	MHP7448_0240	6/6 (100%)	-2.49
glpQ	glycerophosphoryl diester phosphodiesterase	ugpQ	2/2 (100%)	-3.28
ackA<br>mhp510	acetate kinase<br>hypothetical protein	ackA<br>MHP7448_0512	4/4 (100%)	-4.92
atpG<br>tsf	ATP synthase gamma chain<br>elongation factor ts	atpG<br>tsf	2/2 (100%)	-5.61
dnaN	DNA polymerase III, beta chain	dnaN	2/2 (100%)	-3.59
hpt	hypoxanthine phosphoribosyl transferase	hpt	2/2 (100%)	-2.99
mhp006	hypothetical protein	MHP7448_006	5/5 (100%)	-8.06
mhp022	ABC transporter ATP binding protein	MHP7448_020	1/1 (100%)	-1.32
mhp639	hypothetical protein	MHP7448_0620	4/4 (100%)	-4.32
ushA	5'-nucleotidase	MHP7448_0630	7/7 (100%)	-6.04
mhp666	hypothetical protein	MHP7448_0646	3/4 (75%)	-7.42
mhp087	hypothetical protein	MHP7448_079	0/4 (0%)	*
mhp411	hypothetical protein	MHP7448_397	2/2 (100%)	-5.40
metK	S-adenosylmethionine synthetase	MHP7448_451	1/1 (100%)	-9.21
nrdF	ribonucleoside-diphosphate reductase beta chain	nrdF	2/4 (50%)	-4.53
rpl2	50s ribosomal protein L2	rplB	1/1 (100%)	-3.77
rpsL	30s ribosomal protein S12	rpsN	2/2 (100%)	-3.26
smpB	SsrA-binding protein	smpB	2/2 (100%)	-1.16
topA	DNA topoisomerase I (omega-protein)	topA	5/6 (83%)	-7.85
rps19<br>rpl14<br>rpl6<br>rps5<br>rpoA	30s ribosomal protein S19<br>50s ribosomal protein L14<br>50s ribosomal protein L6<br>30s ribosomal protein S5<br>DNA-directed RNA polymerase alpha chain	rpsS<br>rplN<br>rplF<br>rpsE<br>rpoA	6/6 (100%)	-3.82
mhp377	putative lipoprotein	MHP7448_0366	4/5 (80%)	-4.52
mhp401	hypothetical protein	MHP7448_389	1/1 (100%)	-0.34
mhp429	hypothetical protein	MHP7448_411	4/7 (57%)	-5.47
mhp434	hypothetical protein	MHP7448_431	3/5 (60%)	-2.74
atpA<br>mhp478<br>mhp481	ATP synthase alpha chain<br>hypothetical protein<br>hypothetical protein	atpA<br>MHP7448_480<br>MHP7448_483	6/6 (100%)	-3.87
mhp482	hypothetical protein	MHP7448_484	6/6 (100%)	-3.87
mhp284	hypothetical protein	MHP7448_0095	1/4 (25%)	-5.86
mhp236	chromate transport protein, putative	MHP7448_0146	1/4 (25%)	-2.03
mhp350	hypothetical protein	MHP7448_0338	1/4 (25%)	+0.20
mhp077	hypothetical protein	MHP7448_071	1/1 (100%)	-0.32
p146	p146 adhesin like-protein, p97 paralog	MHP7448_663	7/7 (100%)	-4.79
pepP	xaa-pro aminopeptidase	pepP	2/2 (100%)	-3.24

polC	DNA polymerase III, alpha chain	polC	2/2 (100%)	-6.29
mhp405	hypothetical protein	MHP7448_391	7/7 (100%)	-3.98
mhp406	hypothetical protein	MHP7448_392		
ftsZ	cell division protein	MHP7448_393		
mhp461	hypothetical protein	MHP7448_463	5/5 (100%)	-3.83
ktrA	potassium uptake protein	MHP7448_545	5/5 (100%)	-3.89
nagE	pts system, n-acetylglucosamine-specific enzyme II, ABC component	MHP7448_574	0/1 (0%)	-1.76
pdhD	dihydrolipoamide dehydrogenase	pdhD	5/5 (100%)	-3.14
parC	topoisomerase IV subunit A	parC	3/3 (100%)	-4.40
lspA	lipoprotein signal peptidase (prolipoprotein signal peptidase) (signal peptidase II) (spase II)	lps		
mhp057	hypothetical protein	MHP7448_054	4/4 (100%)	-5.84
mhp064	hypothetical protein	MHP7448_061	2/2 (100%)	-5.61
mhp069	hypothetical protein	MHP7448_065	0/1 (0%)	*
mhp071	hypothetical protein	MHP7448_0681	2/2 (100%)	-3.33
lepA	30 kda GTP-binding protein lepA	MHP7448_072		
dnaK	chaperone protein dnaK	dnaK	3/2 (100%)	-4.74
serS	seryl-tRNA synthetase	serS	6/6 (100%)	-10.73
mhp144	putative dehydrogenase	MHP7448_0235	6/6 (100%)	-5.57
mhp145	putative D-ribose-binding protein mutant	rbsB		
mhp148	hypothetical protein	iolE		
iolD	myo-inositol catabolism	iolD		
mhp150	hypothetical protein	MHP7448_0228		
mhp151	hypothetical protein	iolB		
iolC	myo-inositol catabolism	iolC		
rbsA	ribose transport ATP-binding protein	mglA	1/2 (50%)	+0.17
mhp171	ABC transporter ATP binding protein	MHP7448_0210	3/4 (75%)	-2.35
map	methionine amino peptidase	map	6/6 (100%)	-3.82
rps11	30s ribosomal protein S11	rpsK		
rpl17	50s ribosomal protein L17	rplQ		
clpB	ATP-dependent serine proteinase, 24 protein	clpB	4/4 (100%)	-3.90
glpF	glycerol uptake facilitator protein	glpF	1/1 (100%)	-1.91
glpK	glycerol kinase (ATP:glycerol 3-phosphotransferase) (glycerokinase)	glpK	3/4 (75%)	-1.84
mhp289	hypothetical protein	MHP7448_0090	5/5 (100%)	-2.19
mhp275	hypothetical protein, p102 paralog	MHP7448_0105 (P)	0/1 (0%)	*
mhp271	p97 cilium adhesin paralog	MHP7448_0108	3/3 (100%)	-4.65
mhp254	hypothetical protein	MHP7448_0127	1/1 (100%)	-1.39
mhp334	hypothetical protein	MHP7448_0319	5/5 (100%)	-4.34
mhp374	hypothetical protein	MHP7448_0363	1/1 (100%)	-0.25
mhp399	hypothetical protein	MHP7448_386	4/4 (100%)	-6.17
mhp445	hypothetical protein	MHP7448_443	4/5 (80%)	-7.93
mhp479	hypothetical protein	MHP7448_481	6/6 (100%)	-3.87
asnS	asparaginyl-tRNA synthetase	MHP7448_500	2/3 (67%)	-3.02

rplK	50s ribosomal protein L11	rplK	2/2 (100%)	-3.24
rps13	30s ribosomal protein S13	rpsM	2/3 (67%)	-10.66
tpiA	triosephosphate isomerase	tpiA	2/5 (40%)	-3.73
oppC<br>oppB	oligopeptide transport system permease protein<br>oligopeptide transport system permease protein	oppC<br>oppB	5/6 (83%)	-5.62
P46	46kd surface antigen precursor	MHP7448_0513	5/5 (100%)	-5.49
mhp005<br>mhp013<br>rluC	hypothetical protein<br>hypothetical protein<br>ribosomal large subunit pseudouridine synthase	MHP7448_005<br>MHP7448_013<br>rluC	5/5 (100%)	-8.06
acpD	acyl carrier protein phosphodiesterase	acpD-1	4/4 (100%)	-4.83
gatB	glutamyl-tRNA amidotransferase, subunit B	gatB	0/5 (0%)	*
metG	methionyl-tRNA synthetase	metS	7/7 (100%)	-3.98
mhp337	hypothetical protein	MHP7448_0089	2/2 (100%)	-4.18
mhp240	hypothetical protein	MHP7448_0142	0/1 (0%)	-
mhp235	hypothetical protein	MHP7448_0147	1/4 (25%)	-2.03
mhp023	ABC transporter ATP binding protein	MHP7448_021	4/4 (100%)	-3.08
mhp136	hypothetical protein	MHP7448_0244	1/1 (100%)	-3.71
mod	site-specific DNA-methyltransferase (adenine-specific)	MHP7448_0316	0/1 (0%)	-3.11
mhp353	hypothetical protein	MHP7448_0341	1/4 (25%)	-2.12
mhp373	putative transport system permease protein p69	MHP7448_0362	3/5 (60%)	-0.87
mhp393	hypothetical protein	MHP7448_0381	3/5 (60%)	-3.59
mhp310	hypothetical protein	MHP7448_297	0/1 (0%)	*
mhp419	hypothetical protein	MHP7448_404	2/6 (33%)	-1.14
proS	prolyl aminoacyl-tRNA synthetase	proS	2/5 (40%)	-2.12
rpsT	30s ribosomal protein S20	rpsT	2/2 (100%)	-1.39
mhp038	hypothetical protein	VacB	2/2 (100%)	-5.91
mhp465<br>mhp466<br>bcrA<br>mhp468	hypothetical protein<br>hypothetical protein<br>ABC transporter ATP binding protein<br>hypothetical protein	MHP7448_467<br>MHP7448_468<br>MHP7448_469<br>MHP7448_470	4/4 (100%)	-6.01
mgtE	MG2+ ion transporter	mgtE	6/6 (100%)	-2.93
mhp509	hypothetical protein	MHP7448_0713	4/4 (100%)	-4.92
mhp474	hypothetical protein	MHP7448_476	2/2 (100%)	-2.82
mhp486	hypothetical protein	MHP7448_488	4/4 (100%)	-7.02
mhp584	hypothetical protein	MHP7448_567	1/4 (25%)	-0.90
polA	DNA polymerase I	MHP7448_581	6/6 (100%)	-7.98
mhp619<br>mhp620<br>mhp621	hypothetical protein<br>hypothetical protein<br>hypothetical protein	MHP7448_0600<br>MHP7448_0601<br>MHP7448_0602	0/1 (0%)	*
mhp674	hypothetical protein	MHP7448_653	2/3 (67%)	-3.24

[1]Conservation was determined by dividing number of elements found in MHP_232 by number of elements in MHP_7448. [2]ΔG represents the best values found among all palindrome elements conserved between MHP_7448 and MHP_232. Abbreviations: MHP_232 – *M. hyopneumoniae* strain 232; MHP_7448 – *M. hyopneumoniae* strain 7448.
