# Supplementary material for: Repetitive Elements in Mycoplasma hyopneumoniae Transcriptional Regulation
Source: PLoS One. 2016 Dec 22;11(12):e0168626. doi: 10.1371/journal.pone.0168626 (PMC5179023; doi:10.1371/journal.pone.0168626)
Supplement: S7 Table — (PDF) [file pone.0168626.s009.pdf]

Supplementary Table S7: DNA repeats located in UTR regions					
**Gene**	**TSS (+1)[a*]**	**TSS_Gap[b*]**	**Start Codon***	**REP_ID**	**REP_Gap[c]**
sipS	G	59	ATT	palG_E_245	39
sipS				palG_ES_52	2
recA	A	71	TTA	palG_E_252	68
uvrC	A	77	ATG	palG_E_833	66
uvrC				palG_ES_60	19
clpB	T	16	TTA	palG_E_853	12
rpsJ	G	68	ATG	palG_E_333	56
rpsJ				palG_E_892	6
MHP7448_0198	G	137	ATG	palG_E_339	110
MHP7448_0198				palG_E_896	150
MHP7448_0198				palG_S_1197	45
MHP7448_0225	G	87	ATG	palG_E_358	73
MHP7448_0225				palG_E_359	40
MHP7448_0225				palG_E_906	13
MHP7448_0272	A	56	ATC	palG_E_925	30
MHP7448_0360	A	143	ATG	palG_E_474	36
MHP7448_0360				palG_ES_134	3
pgk	A	25	ATG	palG_E_1051	12
MHP7448_0513	G	35	ATG	palG_E_589	36
pyrH	A	16	ATG	palG_E_1077	14
rplJ	A	100	TTG	palG_ES_218	0
rplJ				SSRM_235_L	84
dam	A	14	TTA	palG_E_1119	4
MHP7448_0663	A	34	ATG	palG_E_1145	12

[a]Transcription Start Sites (+1)

[b]Distance (nucleotides) between the TSS and the start codon

[c]Distance (nucleotides) between the REP and the start codon

*Data from Weber et al. 2012
